# Evaluation of Candidate Genes from Orphan FEB and GEFS+ Loci by Analysis of Human Brain Gene Expression Atlases

**DOI:** 10.1371/journal.pone.0023149

**Published:** 2011-08-17

**Authors:** Rosario M. Piro, Ivan Molineris, Ugo Ala, Ferdinando Di Cunto

**Affiliations:** Molecular Biotechnology Center and Department of Genetics, Biology and Biochemistry, University of Torino, Torino, Italy; Rikagaku Kenkyūsho Brain Science Institute, Japan

## Abstract

Febrile seizures, or febrile convulsions (FEB), represent the most common form of childhood seizures and are believed to be influenced by variations in several susceptibility genes. Most of the associated loci, however, remain ‘orphan’, i.e. the susceptibility genes they contain still remain to be identified. Further orphan loci have been mapped for a related disorder, genetic (generalized) epilepsy with febrile seizures plus (GEFS+).

We show that both spatially mapped and ‘traditional’ gene expression data from the human brain can be successfully employed to predict the most promising candidate genes for FEB and GEFS+, apply our prediction method to the remaining orphan loci and discuss the validity of the predictions. For several of the orphan FEB/GEFS+ loci we propose excellent, and not always obvious, candidates for mutation screening in order to aid in gaining a better understanding of the genetic origin of the susceptibility to seizures.

## Introduction

Febrile seizures, or febrile convulsions (FEB), are acute symptomatic seizures that occur in response to fever and represent the most common form of childhood seizures, affecting worldwide between 2 and 14% of infants before five years of age (reviewed in [1]–[4]). Clinically, febrile seizures are often divided in a mostly benign ‘simple’ type (about 60–70% of incidences), consisting of single generalized tonic-clonic seizures of less than 10–15 minutes without focal neurological features, and a ‘complex’ type of prolonged seizures (15 minutes or more) with possible long-term consequences, having focal neurological features or recurrence within the same febrile illness [5]–[7]. FEB is a complex and heterogeneous disorder in which environmental factors play an important role, the most obvious being an immature brain (suggested by the age-specificity) and fever. However, both experimental data on animal models and known familial cases with Mendelian inheritance demonstrate the significant role that genetic factors may play in the etiology of the disease [2], [3]. Several loci have been associated with FEB predisposition and/or genetic (generalized) epilepsy with febrile seizures plus (GEFS+) [8], [9], often with an autosomal dominant mode of inheritance with incomplete penetrance, suggesting a possibly simultaneous involvement of multiple genes [1], [10]. However, so far only few susceptibility genes have been found [11].

Although FEB is considered to be a distinct syndrome with a generally very good prognosis, and not a true epileptic disease, some evidence points towards a higher risk of patients with a FEB history to develop epileptic disorders later in life [1]–[4], [7], [12]. While most prospective studies have found no evidence for this hypothesis, retrospective studies indicate that a high fraction of patients that require surgery for treatment-resistant temporal lobe epilepsy (TLE) have a history of FEB, and large cohort studies have linked a history of FEB to a greater than five-fold increase in epilepsy later in life, although the overall probability of the latter event remains low.

Moreover, mutations in the genes encoding for the voltage-gated sodium channel 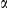-1, 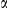-2 and 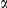-9 subunits ( *SCN1A*, *SCN2A* and *SCN9A*) and the GABA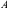 receptor 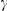-2 subunit gene ( *GABRG2*)–four out of five known FEB susceptibility genes according to the OMIM database (see Table 1)–have been identified in families with GEFS+. In many of the reported cases of familial FEB, at least some of the affected members developed also epileptic seizures [1], suggesting that many FEB loci actually represent genetic loci for GEFS+ [11]. Only the FEB2, FEB5 and FEB8 loci have been reported for pure febrile convulsions [13]–[15] (although the gene for FEB8, *GABRG2*, has also been found mutated in GEFS+ [16]). This may, of course, reflect only the appearance of febrile seizures in GEFS+, as a continuum between febrile and afebrile seizures is typical of GEFS+ [1]. On the other hand, it may also hint at a more profound relation between FEB and true epileptic disorders.

**Table 1:** Genes known to be involved in FEB and/or GEFS+.

Gene	Entrez ID	Phenotype(s)	OMIM ID(s)	Locus	References
*GABRD*	2563	GEFS+5	#604233	1p36.3	[77]
*GABRG2*	2566	FEB8, GEFS+3	#611277, #604233	5q34	[13], [16], [78], [79]
*SCN1A*	6323	FEB3A, GEFS+2	#604403, #604233	2q24.3	[80]–[83]
*SCN1B*	6324	GEFS+1	#604233	19q13.1	[84]
*SCN2A*	6326	FEB/GEFS+	(–)†	2q24.3	[85]
*SCN9A*	6335	FEB3B, GEFS+7	#604403, #604233	2q24	[86]

† *SCN2A* is not listed in OMIM as a FEB or GEFS+ gene, but is usually considered as such [3], [10], [11].

Although the association of FEB with an increased risk of adult epileptic disorders such as TLE remains controversial [1], [17], febrile seizures can lead to epilepsy in some animal models, probably due to an imbalance of excitation and inhibition in the limbic system [4]. Due to the obvious differences between animal models and FEB in humans (see [2], [4] for a discussion), however, the interpretation of the obtained results requires some caution.

On the other hand, direct human studies of the mechanisms underlying FEB have obvious limitations. In this report, we try to aid in elucidating the genetic factors that influence FEB by applying computational disease gene prioritization to evaluate the ‘positional’ candidate genes in previously mapped ‘orphan’ FEB and GEFS+ loci (listed in Table 2), for which the involved susceptibility genes have not yet been determined. We first show that both spatially mapped and ‘traditional’ gene expression data from the human brain can be successfully used to prioritize candidate genes for Mendelian disorders related to the central nervous system (CNS), and that the results obtained for the two data sources are highly complementary. We then demonstrate that this works particularly well for ‘re-discovering’ known FEB/GEFS+ susceptibility genes, suggesting that a prioritization of the candidate genes included in orphan loci may actually yield biologically meaningful predictions. Finally, we apply the method to all orphan FEB and GEFS+ loci and present the most promising candidates. We hope that our predictions may eventually help in gaining a better understanding of the genes that influence the susceptibility to seizures.

**Table 2:** Orphan FEB and GEFS+ loci.

Phenotype	OMIM ID	Locus	Coordinates	Candidates			Ref.
				total	HBA	GEO	
FEB1	%121210	8q13-q21	chr8:66,932,638–72,988,347	31	19	28	[29]
FEB2	%602477	19p13.3	chr19:3,075,844–7,583,551	112	76	105	[14]
FEB4†	#604352	5q14-q15	chr5:89,166,516–110,036,710	50	39	49	[40]
FEB4	#604352	5q14.3-q23.1	chr5:88,728,695–122,125,234	93	70	88	[28]
(Deprez et al.) ^‡^							
FEB5	%609255	6q22-q24	chr6:129,947,710–133,546,636	29	12	27	[15]
FEB6	%609253	18p11.2	chr18:10,133,108–12,597,248	12	11	12	[52]
FEB7	%611515	21q22	chr21:32,533,246–39,359,893	59	43	54	[58]
FEB9	%611634	3p24.2-p23	chr3:24,924,673–33,687,557	32	26	29	[53]
FEB10	%612637	3q26.2-q26.33	chr3:170,902,825–181,419,957	28	24	28	[63]
GEFS+4	%609800	2p24	chr2:17,491,922–20,329,508	13	8	11	[67]
GEFS+6	%612279	8p23-p21	chr8:6,940,747–15,649,945	56	20	36	[72]
GEFS+6	%612279	8p23.1-p21.3	chr8:6,940,747–20,367,401	79	35	59	[72]
(family 15173)*							
GEFS+N	–	6q16.3-q22.31	chr6:101,352,134–119,553,944	89	60	85	[73]

Mapped FEB and GEFS+ loci for which the susceptibility or disease-causing gene has not yet been identified. Coordinates correspond to the regions flanked by genetic markers identified in the respective studies. The number of candidate genes in the mapped regions is indicated as a total number and the subset of genes for which HBA and GEO expression profiles are available, respectively (see Table S4 for the complete lists of candidates). Note: “GEFS+N” is not an official symbol (N = novel).

† The implication of *MASS1* in FEB4 [25] is controverse; Deprez et al [28] describe a case with an overlapping linkage interval (^‡^) without evidence for *MASS1* mutations (see text).

*Baulac et al [72] describe also a family with a larger linkage interval; since it is not clear whether the genetic cause is the same, we consider this interval as a distinct orphan locus.

In our previous proof-of-concept study [18] we have shown that spatially mapped, i.e. 3D, high-resolution gene expression data covering the entire adult brain of the C57BL/6J mouse strain–the anatomically comprehensive Allen Mouse Brain Atlas (MBA) [19]–can be successfully exploited for the prioritization of positional candidates for both mouse phenotypes and human CNS-related hereditary disorders. This particular type of gene expression data is distinct from ‘traditional’ heterogeneous datasets containing samples from multiple tissues and cell types, due to its three-dimensional geometry and tissue-specificity. We have used this dataset to suggest some promising novel candidates for X-linked mental retardation [18].

However, the important differences between human FEB and animal models of the disorder [2], [4], and the fact that differences between mouse models and human brains can yield important insight also for other neurological disorders [20], suggest that an application of our method to the Allen Institute’s recently published Human Brain Atlas (HBA) [21] may be more appropriate, even if the HBA is still in a preliminary version.

In contrast to the more complete MBA, the HBA is not derived from *in situ* hybridization (ISH) images of many different brains, but instead uses a microarray technology on samples from a single individual. It does not yet provide expression data for all consecutive positions of the entire organ since the project is scheduled to be completed in 2013. Nonetheless, the current preliminary version already includes samples for most of the anatomical structures of the human brain.

## Results

### Evaluation for all CNS-related disorders

We evaluated the possibility to predict gene–phenotype associations based on 13,204 HBA-derived 3D gene expression profiles from the human brain using a large-scale leave-one-out cross validation (LOOCV; see Methods) over all CNS-related Mendelian disorders from the Online Mendelian Inheritance in Man (OMIM) database [22] (see Text S1) and compared the results to those previously obtained for the MBA [18].

As in our previous study, we prioritized a list of candidate genes taken from “artificial” orphan loci of various sizes centered around the true phenotype-causing gene 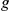 and verified how often 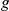 was ranked first (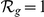), among the top ten (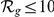), and among the upper 10% (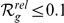) of the candidates. For this purpose, the 3D gene expression profiles of the candidates were compared to those of a set of disease-related reference genes (see Methods).

As can be seen in Table S2, the procedure yielded for all sizes of artificial loci a significantly higher number of positive results than expected by chance, suggesting that spatial gene expression data from the HBA can be an important source of information to predict novel gene–phenotype associations. The HBA expression profiles perform similarly to those of the MBA, in some cases even better. This may seem obvious, given that we consider human disorders, but it is less clear when taking into account that the mouse 3D expression profiles cover the entire brain and have a far higher-resolution than the preliminary human data and that the two brain atlases are based on completely different technologies (ISH vs. microarray).

Better results are obtained when taking the known molecular basis of the disorders as reference (simulating phenotypes with partially known molecular basis), instead of relying on reference genes from similar OMIM phenotypes (to simulate phenotypes with unknown molecular basis; see Text S1). Nonetheless, the results obtained when simulating an unknown molecular basis are highly significant and suggest that the method can be used also for phenotypes to which so far no disease genes have been associated.

### Complementarity of HBA and GEO data

Since the HBA is based on microarray technology, in contrast to the MBA that uses ISH, we asked how the disease gene prioritization method performs using ‘traditional’ microarray data from multiple individuals. For this purpose we compiled a dataset of 453 normal brain samples for 19,946 genes from the Gene Expression Omnibus (GEO) [23].

The GEO dataset performs better than the HBA data (see Table S2). This is likely due to the greater number of genes for which expression profiles are available. Nonetheless, given the different nature of the two datasets the positive predictions derived from both have only a limited overlap (no overlap for 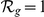 and less than 30% for both 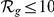 and 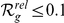; see Table S3). This suggests that the 3D gene expression data–spatially distributed samples from a single individual–provides information that is complementary to that provided by ‘traditional’ expression data–arbitrarily distributed samples from many individuals. Also the fact that different microarray platforms have been used for the two datasets is likely to play a role [24].

Consequently, some cases can be successfully predicted when relying on spatial expression information while others using ‘traditional’ expression data. We therefore suggest that while the best candidates are those derived from an intersection of the prioritizations from the two data sources, those candidates obtained from only one of the datasets should still be taken into consideration. Although the GEO dataset performs generally better, it cannot replace the HBA predictions.

### Evaluation for FEB and GEFS+

The effectiveness of our approach for CNS-related disease phenotypes in general does not necessarily imply that it can be successfully used for FEB and GEFS+ in particular. Febrile seizures are age-dependent and occur in immature brains of infants and young children. It is therefore imperative to specifically evaluate whether in this case expression data from the adult brain, that shows important differences in gene expression patterns, can aid in prioritizing candidate genes.

In order to verify the performance of our approach for the specific case of FEB and GEFS+, we performed an LOOCV taking into account only the reference genes listed in Table 1, found to be associated with FEB and GEFS+ in OMIM and/or the literature. From this list, we excluded *MASS1* ( *GPR98*) that was suggested by Nakayama et al [25] to be the disease gene for the FEB4 locus because the gene had been reported to be involved in audiogenic seizures in the Frings mouse strain [26], [27] and they found a nonsense mutation in a small family with febrile and afebrile seizures. However, the cosegregation of the mutation with the seizure phenotype was not unambiguous due to the small family size. Moreover, another linkage study has mapped FEB/GEFS+ to an overlapping (although larger) locus, but did not find disease-causing mutations in the exons of *MASS1* (or *VLGR1* that includes most *MASS1* exons), suggesting that another gene may be underlying the FEB4 locus [28]. Due to this uncertainty, we chose to consider this linkage interval as an additional orphan locus (see Table 2) and *MASS1* as a candidate, rather than a disease gene.

As for the large-scale LOOCV performed over all CNS-related disorders, we constructed artificial loci of various sizes centered around each of the FEB or GEFS+ genes and took the remaining five genes as reference genes for the prioritization.

Table 3 demonstrates that in most of the prioritizations the true FEB/GEFS+ gene is ranked among the top ten and/or the best 10% of the candidates from the artificial locus. Most important, four of the six genes ( *GABRD*, *GABRG2*, *SCN1B*, and *SCN2A*) rank in at least one of the two datasets among the first three candidates for artificial loci of similar sizes (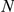 = 50) as the orphan loci (between 12 and 112 genes, see Table 2). Even for far larger loci of up to 401 candidates (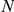 = 200) half of the disease genes have 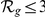, suggesting that the method may be particularly effective for FEB and GEFS+.

**Table 3:** Leave-one-out cross-validation of known FEB and GEFS+ genes.

Gene 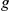	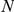	HBA		GEO	
		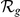	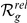	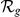	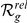
*GABRD*	50	**3** 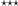	0.0435	**2** 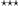	0.0217
	100	**5** 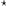	0.0467	**3** 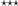	0.0210
	200	**6** 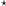	0.0390	**3** 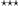	0.0140
	400	**10** 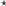	0.0357	**4** 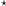	0.0101
*GABRG2*	50	**3** 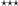	0.0455	**4** 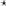	0.0421
	100	**4** 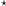	0.0294	**4** 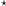	0.0214
	200	**6** 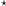	0.0287	**4** 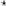	0.0134
	400	**9** 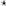	0.0257	**4** 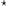	0.0082
*SCN1A*	50	12	0.1644	**4** 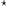	0.0404
	100	28	0.2137	**6** 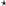	0.0319
	200	42	0.1615	**6** 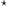	0.0164
	400	72	0.1426	**9** 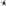	0.0126
*SCN1B*	50	**3** 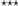	0.0448	**1** 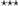	0.0105
	100	**8** 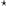	0.0541	**1** 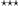	0.0054
	200	**18**	0.0629	**3** 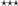	0.0082
	400	**39**	0.0721	**5** 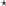	0.0069
*SCN2A*	50	**7** 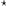	0.1000	**2** 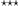	0.0202
	100	**8** 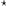	0.0611	**3** 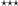	0.0160
	200	**10** 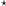	0.0385	**3** 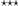	0.0082
	400	**12**	0.0237	**3** 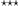	0.0042
*SCN9A*	50	8 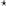	0.1111	65	0.6566
	100	**12**	0.0923	118	0.6277
	200	**20**	0.0769	230	0.6284
	400	**27**	0.0536	459	0.6411

Absolute (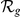) and relative (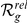) rankings of the known FEB and GEFS+ genes 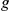 (see Table 1) for LOOCVs using different artificial locus sizes 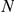 (up to 2
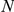
+1 genes). Results are shown for both gene expression datasets, HBA and GEO. Ranks among the best 10% (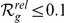) are evidenced by bold face font. Ranks among the top 10 (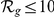) are additionally marked by a single star (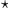) and ranks among the top 3 (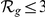) by three stars (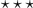).

Although, as for the large-scale LOOCV, the GEO expression data generally performs better, in some cases the rankings obtained with the HBA data are better. Interestingly, this is the case for *SCN9A* that does not show a particularly strong relationship with the other reference genes, but whose involvement in FEB/GEFS+ is significantly better recalled when applying spatially mapped expression profiles.

### Prediction for orphan FEB and GEFS+ loci

Given the good performance of our prioritization procedure, as demonstrated by the CNS-related and the GEFS+/FEB-specific LOOCVs, we processed all orphan disease loci (see Table 2) to find the most promising candidates for an involvement in FEB or GEFS+ susceptibility using only the known disease genes (Table 1) as reference genes.

Since we found the HBA and GEO expression data to be complementary for the purpose of disease gene prediction for CNS-related disorders, in Table 4 we report for all orphan loci the best ranking candidate for each of the two data sources, as well as the candidate that has the best average relative rank (average of 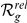) over both datasets. For the complete prioritized candidate lists that contain further interesting candidates, please see Table S4.

**Table 4:** Prediction results for orphan FEB and GEFS+ loci.

Phenotype /	Candidate		HBA		GEO		average
Orphan locus	Entrez ID	Symbol	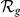	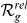	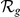	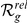	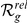
FEB1	10565	*ARFGEF1*	**1**	0.0526	19	0.6786	0.3656
FEB1	80124	*VCPIP1*	3	0.1579	**1**	0.0357	**0.0968**
FEB2	56961	*SHD*	**1**	0.0132	8	0.0762	0.0447
FEB2	60680	*BRUNOL5*	3	0.0395	**1**	0.0095	0.0245
FEB2	79187	*FSD1*	2	0.0263	2	0.0190	**0.0227**
FEB4	2745	*GLRX*	**1**	0.0256	10	0.2041	0.1149
FEB4	5066	*PAM*	17	0.4359	**1**	0.0204	0.2282
FEB4	1946	*EFNA5*	4	0.1026	2	0.0408	**0.0717**
FEB4 (Deprez et al.)	9315	*C5orf13*	**1**	0.0143	12	0.1364	0.0753
FEB4 (Deprez et al.)	5066	*PAM*	33	0.4714	**1**	0.0114	0.2414
FEB4 (Deprez et al.)	814	*CAMK4*	4	0.0571	2	0.0227	**0.0399**
FEB5	9465	*AKAP7*	**1**	0.0833	7	0.2593	0.1713
FEB5	114801	*TMEM200A*	3	0.2500	**1**	0.0370	0.1435
FEB5	285735	*LOC285735*	-	-	2	0.0741	**0.0741**
FEB6	8774	*NAPG*	**1**	0.0909	**1**	0.0833	**0.0871**
FEB7	3763	*KCNJ6*	**1**	0.0233	2	0.0370	**0.0301**
FEB7	7074	*TIAM1*	20	0.4651	**1**	0.0185	0.2418
FEB9	7342	*UBP1*	**1**	0.0385	2	0.0690	**0.0537**
FEB9	25827	*FBXL2*	10	0.3846	**1**	0.0345	0.2095
FEB10	5290	*PIK3CA*	**1**	0.0417	3	0.1071	0.0744
FEB10	57552	*AADACL1*	2	0.0833	**1**	0.0357	**0.0595**
GEFS+4	7447	*VSNL1*	**1**	0.1250	**1**	0.0909	**0.1080**
GEFS+6	157627	*LOC157627*	**1**	0.0500	**1**	0.0278	**0.0389**
GEFS+6 (family 15173)	286097	*EFHA2*	**1**	0.0286	2	0.0339	**0.0312**
GEFS+6 (family 15173)	157627	*LOC157627*	2	0.0571	**1**	0.0169	0.0370
GEFS+N	7259	*TSPYL1*	**1**	0.0167	**1**	0.0118	**0.0142**

For each orphan FEB or GEFS+ locus (see Table 2) we report the best ranking candidate gene for HBA expression data, for GEO expression data and the candidate gene with the best average 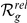 (indicated by bold face font). For the complete prioritized candidate lists, see Table S4. 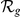  =  absolute rank; 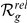  =  relative rank (among candidates with expression profiles). Note: “GEFS+N” is not an official symbol (N = novel).

Strikingly, for most of the orphan loci, the results obtained with HBA and GEO data support each other. Indeed, in the large majority of cases, the first ranking candidate of one data source often obtained a high rank also for the other data source and vice versa, so that one of the two first ranking candidates (from HBA or GEO) remains the best candidate also on average. Only for four of the thirteen loci (FEB2, both FEB4 loci, and FEB5) the best average candidate did not rank first for HBA or GEO. Most important, for several of the loci (FEB6, GEFS+4, GEFS+6, and GEFS+N), both spatially mapped and traditional expression data agree upon the best candidate. This result is especially remarkable for the GEFS+N locus, being the third largest orphan locus with a total of 89 candidates (see Table 2).

## Discussion

Overall, the good agreement between the prioritizations for FEB/GEFS+ obtained starting from HBA and GEO expression data increases the likelihood that the best candidates are functionally correlated to known FEB and GEFS+ genes and thus potentially disease relevant. Therefore, in the following sections we discuss particularly interesting candidates for the known orphan loci.

### FEB1

The first FEB locus was mapped by Wallace et al [29] in a large Australian family spanning three generations. Results supported a hypothesis of autosomal dominant inheritance with incomplete penetrance, i.e. conditional upon the involvement of other modifier loci or environmental factors.

Wallace et al [29] have proposed corticotropin-releasing hormone (CRH) as one of the most interesting candidates of this locus. Indeed, in our prioritizations, *CRH* ranks 4th for the HBA expression data and 2nd for GEO, as well as 2nd with respect to the average relative rank over both datasets (see Table S4). CRH is known to be involved in an age-dependent manner in seizures in rat models [30], [31] and its activation is thought to be an important mechanism for generating developmentally regulated, triggered seizures [32]. Notably, some studies support a region-specific regulation of *CRH* gene expression by gamma-aminobutyric acid (GABA). Tran et al [33], for example, showed that the GABA degradation blocker gamma-vinyl-GABA (VGB) that is used for clinical seizure treatment causes a reduction of both CRH gene expression and secretion in the hypothalamus of 9-day-old rats. Finally, an excess in CRH has been hypothesized to underlie West syndrome, in which seizures during infancy play an important role [34], and CRH has been found to play a role in the increased susceptibility to seizures in shigellosis [35].

Taken together, *CRH* is a likely candidate for this locus, and a mutation that causes an increase in CRH levels or activity would be consistent with an autosomal dominant inheritance. Our two first ranking candidates *ARFGEF1* and *VCPIP1*, instead, have so far not been implicated in seizures or epileptic disorders, but their coexpression with the reference genes in the human brain suggests a potential functional relationship.

### FEB2

Johnson et al [14] mapped the FEB2 locus in a large multi-generational family with an autosomal dominant inheritance of febrile convulsions.

A potentially interesting candidate for the largest FEB/GEFS+ orphan locus is the *ATCAY* gene, which has been found mutated in human Cayman ataxia [36] and in a form of rat dystonia [37]. This gene ranks 5th for the HBA expression data and 3rd for GEO, as well as 4th with respect to the average relative rank over both datasets (see Table S4). *ATCAY* encodes for BNIP-H or Caytaxin, a brain-specific member of the BNIP-2 family that reduces the steady-state levels of glutamate by inhibiting kidney-type glutaminase (KGA) enzyme activity, affecting glutamate synthesis at synapses during neurotransmission [38]. Loss of function of *ATCAY* may lead to deregulated glutamatergic activation or even to glutamate excitotoxicity [38]. Hence, we propose that a mutation of the *ATCAY* gene may potentially lead to an imbalance between excitation and inhibition that increases the susceptibility to seizures.

The coexpression of *BRUNOL5*, or *CELF5*, with the reference genes is also intriguing. Indeed, this gene encodes for a brain-specific splicing factor (although its function is likely not limited to the regulation of alternative splicing) [39] that may in theory affect the splicing of other genes related to FEB/GEFS+.

### FEB4

We have considered two different linkage intervals for FEB4, since Deprez et al [28] mapped a locus that overlapped the one first identified by Nakayama et al [40], who had suggested *MASS1* ( *GPR98*) as the disease causing gene [25]. The evidence, however, was not conclusive and Deprez et al [28] did not find any disease-causing mutations in the exons of *MASS1* (see above), so that we considered it as a regular candidate.

In our prioritization for the original FEB4 locus, *GPR98* ranks 16th and 20th for HBA and GEO expression data, respectively. In the larger locus identified by Deprez et al [28] the ranks are even less suggestive (31st for HBA and 39th for GEO, see Table S4). Hence, at least with respect to expression profiles in the human brain, as compared to those of the reference genes, *MASS1* does not seem to be a particularly strong candidate. Of course this result does not imply that this gene is not involved, as we do not expect all disease genes to be revealed by a coexpression-based approach. Its involvement in audiogenic seizures in the Frings mouse strain [26], [27] remains a strong argument for a possible implication in FEB/GEFS+.

However, among the other candidates found in these overlapping loci, we think that the peptidylglycine 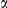-amidating monooxygenase ( *PAM*) gene is a particularly promising alternative. Indeed, although *PAM* is not a very strong candidate with the HBA dataset, it is the top scoring candidate in both intervals with the GEO dataset. The protein encoded by the *PAM* gene is a cuproenzyme essential for the synthesis of many neuropeptides [41]. Finally, and quite remarkably, it has recently been shown that mice heterozygous for a null mutation in the *PAM* gene are more susceptible to chemically induced seizures [42]. Taken together, these evidences point to *PAM* as a very strong candidate for FEB4.

But also *GLRX* and *C5orf13* are potentially interesting candidates. Glutaredoxin (thioltransferase), or GLRX, regulates the activity of the copper-transporting P-type ATPases ATP7A and ATP7B [43] that are involved in Menkes disease (OMIM: #309400) and Wilson disease (OMIM: #277900), respectively. Since epilepsy is a major feature of Menkes disease [44], [45], and seizures occur at low frequency in patients affected by Wilson disease [46], [47], it is conceivable that reduced function of *GLRX* may lead to increased neuronal excitability. On the other hand, *C5orf13* (also *PTZ17* or *P311*) lies outside of the original FEB4 locus but might be a candidate for the larger locus reported by Deprez et al [28], since it was identified as differentially expressed after inducing seizures in mice [48]. However, no seizures were reported in *C5orf13* knockout mice [49].

### FEB5

Nabbout et al [15] suggested that the locus mapping to 6q22-q24 was the first locus identified for pure simple febrile seizures, the most frequent form of FEB. They excluded the implication of several candidate genes, such as syntaxin 7 ( *STX7*), A kinase anchor protein 7 ( *AKAP7*, also *AKAP18*), putative neurotransmitter receptor ( *PNR*), G protein receptor 58 ( *GPR58*) and G protein receptor 57 ( *GPR57*) by sequencing their coding exons and exon–intron boundaries.

According to our analysis, *AKAP7* is one of the best candidates, ranking 1st for the HBA. AKAP7, that is expressed among other tissues in the cerebral cortex, targets the cAMP-dependent protein kinase (PKA) to the plasma membrane, permitting its functional coupling with L-type calcium channels [50]. These voltage-gated channels control a variety of neuronal functions that are implicated in epileptogenesis [51]. It is in principle possible that the affected family members studied by Nabbout et al [15] are carriers of a mutation that effects regulatory elements or other essential non-coding residues of *AKAP7*. Hence, we suggest not to exclude the possibility that a mutation in *AKAP7* is the true cause for the FEB5 locus.

### FEB6

The haplotype analysis performed by Nakayama et al [52] indicated a possible role for the *IMPA2* gene, which is located in the FEB6 locus. Moreover, Nabbout et al [53] have found a locus on chromosome 18p to contain a modifier gene who's cosegregation with the FEB9 locus on 3p (see FEB9) is associated with more severe disorders like childhood absence epilepsy (CAE) and TLE, but is likely to also contribute to febrile seizures per se [53], suggesting that the modifier locus could actually be the FEB6 locus. Thus, they sequenced the *IMPA2* gene as most likely candidate, but found no disease-causing mutations. In our analysis, *IMPA2* has no significant rankings (5th out of 11 for HBA and 8th out of 12 for GEO expression data). However, neither the lack of mutations in [53] nor our negative result represent sufficient evidence to exclude this candidate.

The best candidate identified by our approach is the N-ethylmaleimide-sensitive factor attachment protein gamma ( *NAPG*) that ranks first with both the HBA and the GEO datasets. The encoded protein belongs to the SNAP family, a group of factors involved in membrane fusion events by allowing NSF proteins to target membranes. Accordingly, the NAPG protein has been shown to mediate platelet exocytosis and to control the membrane fusion events of this process [54]. Although the gene has never been studied in a neuronal context, genetic studies suggest that it could play an important role in neurons, because a possible involvement in bipolar disorders has been detected by several independent reports [55]–[57].

### FEB7

The orphan FEB7 locus was mapped by Hedera et al [58] in a five-generation family with autosomal dominant febrile seizures. Of 13 individuals affected by FEB, three showed coexisting afebrile seizures. The authors sequenced all exons of four ion-channel genes–the potassium channel genes *KCNE1*, *KCNE2* and *KCNJ6* and the intracellular chloride channel gene *CLIC6*–but did not identify any disease-causing mutations. Three of these candidates ( *KCNE1*, *KCNE2* and *CLIC6*) have no HBA expression profiles available, but rankings obtained with the GEO dataset are rather low (37th, 26th and 36th out of 54, respectively). *KCNJ6*, however, was ranked 1st for the HBA expression data and 2nd for GEO. As for *AKAP7* and FEB5, a mutation in a regulatory element or in another essential non-coding region should not be excluded.

On the other hand, even the *TIAM1* gene, that ranks first with the GEO dataset, could be an interesting candidate, because studies conducted in rodents have shown that the encoded protein plays a role in axonogenesis [59], in neuronal migration [60] and in the formation of dendritic spines [61].

### FEB9

Nabbout et al [53] mapped the FEB9 locus in a large French family with febrile seizures but also CAE and TLE that were largely associated with an additional modifier locus on chromosome 18p (see FEB6). The authors have sequenced the coding exons, exon-intron boundaries and translation start sites of six potential candidates of the FEB9 locus without finding disease-causing mutations. Of these, the *SCL4A7* gene encoding the sodium bicarbonate cotransporter NBC3 scored best in our prioritizations (3rd for HBA, 10th for GEO and 5th for its average relative rank).

The two candidates that rank first for HBA and GEO data, respectively, are difficult to link to FEB or GEFS+.

However, *GPD1L*, which ranks 5th for HBA, 3rd for GEO, and 3rd with respect to its average relative rank, encodes the glycerol phosphate dehydrogenase 1-like protein, an enzyme that may modify heart excitability by regulating the activity of the sodium channel SCN5A through PKC-dependent phosphorylation [62]. *SCN5A* itself was one of the candidates sequenced by Nabbout et al [53] (although it lies for about 5-Mb outside of the FEB9 locus), but had no causal mutations. It is in principle thinkable that *GPD1L* instead may be responsible for the phenotype through its capability to regulate the activity of SCN5A or other sodium channels in the brain, some of which have already been implicated in FEB/GEFS+ (see Table 1).

### FEB10

Dai et al [63] mapped the FEB10 locus in a four-generation Chinese family with autosomal dominant febrile seizures and epilepsy. By mutational analysis, they have excluded the two potassium channel genes *KCNMB2* and *KCNMB3*, none of which ranks among the top five in our analysis (average relative rank over HBA and GEO).

The strongest candidates identified by our analysis are *AADACL1* and *PIK3CA* that rank very favorably with both datasets. The involvement of the first gene is not supported by other evidences, because AADACL1, also known as NCHE1 (neutral cholesterol ester hydrolase 1), has never been studied in a neuronal context. On the other hand, PIK3CA, the p110 alpha subunit of PI3K, which is known mostly for its role in growth factor-activated signaling and for its oncogenic potential, has recently been shown to negatively regulate neuronal excitability [64]. In addition, levels of phosphorylated (inactive) FOXO, a product of PI3K/Akt activity, are strongly modulated in experimental models of epilepsy and in the hippocampi of epileptic patients [65]. Finally, it has been recently observed that application of leptin, a known PI3K activator, inhibits seizures in rats in a PI3K-dependent manner [66]. Thus, it is conceivable that mutations leading to reduced PI3K activity might be a cause of increased neuronal excitability, possibly leading to febrile seizures.

### GEFS+4

Audenaert et al [67] have suggested that *KCNS3* (voltage gated potassium channel, subfamily S, member 3) is one of the most attractive candidate genes in the GEFS+4 locus, since it encodes a subunit of the voltage-gated potassium channel of the delayed rectifier type and is abundantly expressed in the brain [68]. We find this gene ranking 3rd for HBA and 6th for GEO (5th with respect to its average relative rank), but given the small locus size, these ranks cannot be deemed as very significant.

According to our prioritization, we propose *VSNL1* (also *VILIP-1*) as a potential alternative candidate. Indeed, besides ranking first for both the HBA and the GEO dataset, this gene may be strongly involved in regulating neuronal excitability. VSNL1 is a member of the visinin/recoverin subfamily of neuronal calcium sensor proteins [69]. Interestingly, it has been recently found that VSNL1 may lead to the upregulation of functional 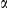
4
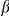
2 nicotinic acetylcholine receptors (nAChR) in hippocampal neurons [70]. This observation could be very important, because a dysfunction of the 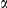
4
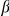
2 nAChR has been previously implicated in frontal lobe epilepsy [71]. Therefore, a mutation that should result in increased function of VSNL1 would be predicted to produce increased neuronal excitability.

### GEFS+6

The locus for GEFS+6 was mapped by Baulac et al [72] in two independent French families. More precisely, the largest family (family 15173) showed a linkage to a 13-Mb interval between markers D8S1706 and D8S258 (chr8:6,840,747-20,367,401), but since a second family showed a linkage to an overlapping region, the authors assumed that in both families the same gene was affected and hence narrowed to region down to a 7.3-Mb candidate interval (chr8:6,940,747-15,649,945).

However, it is in principle possible that the genetic causes are distinct for the two families and hence we have performed the prioritization for both intervals. The results of these prioritizations are in good agreement, since the top three genes (with respect to average relative rank) for the narrow locus– *LOC157627*, *MTMR9*, *PRAGMIN*–rank 2nd, 4th, and 3rd for family 15173, respectively (see Table S4). *LOC157627* is the best ranking gene for GEO in both the narrowed and the larger locus. *EFHA2*, although lying for about 1.2-Mb outside of the narrowed GEFS+6 locus, has particularly good rankings for the locus of family 15173 (1st for HBA, 2nd for GEO, and 1st regarding the average relative rank) and might thus be taken as an additional interesting candidate.

Baulac et al [72] have sequenced the coding exons of six candidates– *MTMR9*, *MTMR7*, *CTSB*, *SGCZ*, *SG223* ( *PRAGMIN*), and *ATP6V1B*–according to their expression in the brain and putative function, but all identified variants were either known polymorphisms or found in 100 matched French controls. Two of these candidates are suggested as interesting by our analysis: *MTMR9* and *PRAGMIN* (see Table S4). Their rankings for the larger locus of family 15173 are less suggestive, but still good. It is in principle possible that mutations in their regulatory elements or other essential non-coding regions are causative for GEFS+. Therefore, we suggest not to exclude them from further studies.

### GEFS+N

Not having found an official denomination, we called this locus “GEFS+N” (where N stands for ‘novel’). The locus contains no known genes associated with ion channels or neurotransmitter receptors, therefore Poduri et al [73] suggest that the identification of the responsible gene may lead to novel insight into the mechanisms of febrile seizures and inherited epilepsy. This makes our prediction results for this locus particularly interesting, but also more difficult to interpret, since a coexpression with known disease genes (reference genes) is more likely if a candidate is involved in a similar function.

Of the 16 candidates sequenced and excluded by Poduri et al [73], *KPNA5* obtained the highest average relative rank in our prioritization (9th for HBA, 11th for GEO, 7th with respect to the average relative rank).

The *TSPYL1* and *TSPYL4* genes come out as outstanding candidates from our analysis. Indeed, they are found as the first and second ranking genes, respectively, for both the HBA and GEO dataset; a very strong result if considering that the locus contains a total of 89 candidates. The two genes are closely related members of the Nucleosome Assembly Protein (NAP) family. Both genes have been investigated as candidates for a genetic syndrome characterized by sudden infant death from cardiac and respiratory arrest, associated with dysgenesis of the testes, and null mutations have been found in *TSPYL1* [74]. However, the molecular mechanism leading to this phenotype is not understood. Therefore, we think that it would be very interesting to evaluate both genes for mutations in GEFS+N. Moreover, it could be worth addressing whether increased or decreased function of these genes may affect the electrical behavior of excitable cells, such as neurons and cardiomyocytes. These studies could contribute to elucidating the mechanisms underlying both febrile seizures and sudden death syndrome.

### Conclusions

In this study, we have shown that the preliminary Human Brain Atlas (HBA), although not yet complete, is already a powerful tool for disease gene prediction and provides results that are complementary to those obtained from a traditional microarray dataset.

Despite the fact that the HBA and GEO gene expression data represent mostly the adult brain and not the immature brain, an application to FEB/GEFS+ seemed particularly promising, given that, in an LOOCV with artificial loci of approximately the same size as the largest FEB or GEFS+ orphan loci, four out of six known disease genes ranked among the first three candidates for at least one of the two human brain gene expression atlases. This allowed us to propose several strong candidates for the true orphan loci and to discuss their possible involvement in FEB/GEFS+. Several of them would be excellent candidates for mutation screenings, however also high ranking candidates that are less obviously related to neuronal excitability should not be excluded from further analysis. In fact, the purpose and strength of computational approaches like the one presented here is to potentially uncover so far unknown and non obvious functional relationships.

## Methods

### Spatial human brain gene-expression data

We downloaded the spatial gene-expression data of the Human Brain Atlas (HBA) from [21] on 20 July 2010, but used only the most recent experiment for each tissue ID, resulting in a total of 798 tissue samples. After normalization with the “Agi4x44PreProcess” Bioconductor R package, we mapped the probesets of the custom Agilent 8x60 Whole Human Genome array (that is derived from the standard Agilent 4x44 Whole Human Genome array) to Entrez gene IDs via the RefSeq RNA nucleotide accessions reported in the downloaded data files. We considered only unique mappings to Entrez IDs and averaged expression profiles of genes with multiple probesets, obtaining expression profiles for a total of 13,204 genes.

### Traditional microarray expression data

For comparison with the spatially mapped gene expression data we compiled a microarray dataset from GEO [23], containing 453 samples of normal human brain tissue (excluding tumor samples or other diseases) performed on a standard Affymetrix HG-U133 Plus 2.0 chip. We chose the Affymetrix platform over the Agilent 4x44 because for the latter only four normal brain samples were available. A list of the GEO accessions of all samples can be found in Table S1. We normalized the dataset with the Affymetrix MAS5.0 algorithm and mapped probeset IDs to Entrez IDs using the official Affymetrix annotation (na30), but accepted only probesets with unique Entrez IDs (19,946 genes). For genes with multiple probesets, we used averaged expression profiles.

### Candidate gene prioritization

The evaluation and prioritization of positional candidates was performed as in our previous study. The following is a brief summary of the procedure, for a more detailed description, please see [18].

First, a set of ‘reference genes’ is defined as the genes that are involved in the given phenotype or in similar phenotypes (see Text S1). Then, for each reference gene, all other genes are ranked in a genome-wide coexpression list according to their decreasing Pearson correlation coefficient with the reference gene.

The relative ranks of the positional candidates within these reference coexpression lists are determined (i.e. the ranks divided by the number of genes in the lists) and overall scores for the single candidates are determined as the product of their relative ranks.

Finally, positional candidates are sorted, i.e. prioritized, according to their increasing overall scores, since lower scores indicate a higher correlation with the reference genes and thus a higher probability of being functionally related to (and hence more likely involved in) the given phenotype.

### Leave-one-out

We performed large-scale LOOCVs over all CNS-related Mendelian disorders for both the spatially mapped expression data from the HBA and the GEO dataset. For each known gene–disease association, we constructed an artificial locus comprising the disease gene itself and the 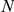 closest genes on both sides of the chromosome (for 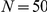, 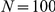, 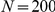, and 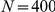), hence containing at the most 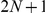 genes. Then, we prioritized the candidates from the artificial loci and verified the absolute rank (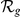) and relative rank (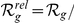
number of candidates) of the true disease gene 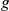 within the prioritized candidate list. For more details, please see Supporting Text S1.

### Candidate gene lists

To identify candidate genes (in terms of Entrez gene IDs) residing between or at the markers flanking the mapped linkage intervals, reported in the respective publications (see Table 2), we used the UCSC Genome Browser [75].

## Supporting Information

Table S1 **List of GEO accessions of the ‘traditional’ microarray expression data set.**
(PDF)

Table S2
**Results of the leave-one-out tests for human CNS-related OMIM phenotypes.** Legend: 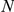 represents the size of the artificial loci having a maximum of 2
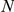
+1 genes. The average numbers of effective candidates with expression profiles and the numbers of evaluated 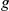
–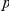 pairs are shown. The observed and expected numbers of 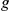
–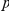 pairs, for which the true phenotype-causing gene 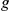 ranks first, among the top ten and within the best 10% of the prioritized list, is reported along with the corresponding 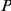-values (one-tailed Fisher exact test). Significant 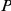-values are highlighted (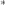 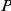 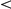
0.05; 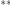 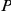 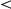
0.01; 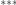 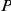 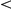
0.001). Reference genes (Ref.) were either taken from similar phenotypes (sim.), or from the OMIM disease phenotype itself (dis.). 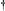
Results taken from [18]; artificial loci with 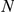 = 400 have not been evaluated in our previous study. (PDF)

Table S3
**Overlap of correct leave-one-out predictions (over all CNS-related OMIM phenotypes) for reference genes from similar phenotypes.** Legend: 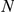 represents the size of the artificial loci, having a maximum of 2
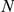
+1 genes. The number of predictions with the true phenotype-causing gene 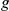 ranking first, among the top ten and within the best 10% of the prioritized list (see also Table S2, is reported for the Human Brain Atlas (HBA) and the GEO microarray dataset (GEO) along with their overlap. Parentheses indicate the corresponding fraction of the maximum possible overlap. (PDF)

Table S4
**Candidate gene prioritizations/predictions for orphan FEB and GEFS+ loci.** Legend: locus  =  orphan FEB/GEFS+ locus; candidate_id/ symbol  =  Entrez gene ID and symbol of the positional candidate; rank_HBA/ GEO  =  rank among the prioritized positional candidates for the HBA/GEO dataset; relRank_HBA/GEO  =  relative rank among the prioritized positional candidates for the HBA/GEO dataset (rank divided by number of candidates with expression data); avRelRank_HBA+GEO  =  average relative rank, i.e. 
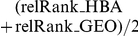
 if the candidate has expression data in both datasets, otherwise the average relative rank is taken as either relRank_HBA or relRank_GEO if only one dataset provides expression data, or remains undefined if no expression data is available. (XLS)

Text S1 **Supplementary Methods.** Definition of CNS-related Mendelian disorders; similarity of human disease phenotypes; leave-one-out cross validation.
(PDF)
