# Supplementary material for: Evaluation of Candidate Genes from Orphan FEB and GEFS+ Loci by Analysis of Human Brain Gene Expression Atlases
Source: PLoS One. 2011 Aug 17;6(8):e23149. doi: 10.1371/journal.pone.0023149 (PMC3157479; doi:10.1371/journal.pone.0023149)
Supplement: Table S1 — List of GEO accessions of the ‘traditional’ microarray expression data set. (PDF) [file pone.0023149.s001.pdf]

Evaluation of candidate genes from orphan FEB and GEFS+ loci by analysis of human brain gene expression atlases

Rosario M. Piro, Ivan Molineris, Ugo Ala
and Ferdinando Di Cunto

Table S1

List of GEO accessions of the 'traditional' microarray expression data set.

GSM80565	GSM80566	GSM80567	GSM80568	GSM80569	GSM80570
GSM80571	GSM80572	GSM80573	GSM80574	GSM80575	GSM80581
GSM80585	GSM80586	GSM80587	GSM80591	GSM80592	GSM80593
GSM80594	GSM80595	GSM80596	GSM80597	GSM80598	GSM80599
GSM80600	GSM80601	GSM80616	GSM80617	GSM80618	GSM80619
GSM80620	GSM80621	GSM80622	GSM80623	GSM80626	GSM80627
GSM80628	GSM80636	GSM80637	GSM80638	GSM80639	GSM80640
GSM80641	GSM80642	GSM80643	GSM80644	GSM80645	GSM80646
GSM80647	GSM80650	GSM80651	GSM80652	GSM80653	GSM80660
GSM80661	GSM80662	GSM80663	GSM80664	GSM80665	GSM80666
GSM80667	GSM80668	GSM80669	GSM80670	GSM80671	GSM80675
GSM80676	GSM80677	GSM80678	GSM80679	GSM80680	GSM80681
GSM80682	GSM80683	GSM80684	GSM80690	GSM80691	GSM80692
GSM80693	GSM80699	GSM80700	GSM80701	GSM80702	GSM80703
GSM80704	GSM80705	GSM80706	GSM80708	GSM80709	GSM80711
GSM80713	GSM80714	GSM80715	GSM80721	GSM80722	GSM80723
GSM80724	GSM80744	GSM80745	GSM80746	GSM80747	GSM80752
GSM80754	GSM80756	GSM80760	GSM80761	GSM80762	GSM80763
GSM80766	GSM80767	GSM80772	GSM80773	GSM80774	GSM80775
GSM80800	GSM80801	GSM80802	GSM80803	GSM80804	GSM80817
GSM80818	GSM80819	GSM80827	GSM80828	GSM80829	GSM80830
GSM80831	GSM80832	GSM80833	GSM80834	GSM80835	GSM80836
GSM80837	GSM80838	GSM80839	GSM80840	GSM80841	GSM80846
GSM80847	GSM80848	GSM80849	GSM80850	GSM80851	GSM80852
GSM80854	GSM80855	GSM80856	GSM80857	GSM80858	GSM80859
GSM80860	GSM80861	GSM80862	GSM80863	GSM80870	GSM80871
GSM80872	GSM80873	GSM80879	GSM80880	GSM80881	GSM80892
GSM80893	GSM80894	GSM80896	GSM80907	GSM80908	GSM80909

(continued on the next page)

Table S1 (continued)

GSM80910	GSM119615	GSM119616	GSM119617	GSM119618	GSM119619
GSM119620	GSM119621	GSM119622	GSM119623	GSM119624	GSM119625
GSM119626	GSM119627	GSM119628	GSM119629	GSM119630	GSM119631
GSM119632	GSM119633	GSM119634	GSM119635	GSM119636	GSM119637
GSM119638	GSM119639	GSM119640	GSM119641	GSM119642	GSM119643
GSM119644	GSM119645	GSM119646	GSM119647	GSM119648	GSM119649
GSM119650	GSM119651	GSM119652	GSM119653	GSM119654	GSM119655
GSM119656	GSM119657	GSM119658	GSM119659	GSM119660	GSM119661
GSM119662	GSM119663	GSM119664	GSM119665	GSM119666	GSM119667
GSM119668	GSM119669	GSM119670	GSM119671	GSM119672	GSM119673
GSM119674	GSM119675	GSM119676	GSM119677	GSM119678	GSM119679
GSM119680	GSM119681	GSM119682	GSM119683	GSM119684	GSM119685
GSM119686	GSM119687	GSM119688	GSM175829	GSM175830	GSM175831
GSM175832	GSM175842	GSM175843	GSM175844	GSM175845	GSM175846
GSM175847	GSM175848	GSM175849	GSM175850	GSM175851	GSM175852
GSM175853	GSM175854	GSM175855	GSM175856	GSM175857	GSM175858
GSM175859	GSM175860	GSM175861	GSM175862	GSM175863	GSM175864
GSM175869	GSM175870	GSM175871	GSM175872	GSM175873	GSM175874
GSM175875	GSM175876	GSM175877	GSM175885	GSM175886	GSM175887
GSM175888	GSM175893	GSM175894	GSM175895	GSM175901	GSM175902
GSM175903	GSM175904	GSM175907	GSM175956	GSM175957	GSM175958
GSM175959	GSM175987	GSM175988	GSM175989	GSM175990	GSM176017
GSM176018	GSM176020	GSM176024	GSM176030	GSM176031	GSM176033
GSM176034	GSM176036	GSM176037	GSM176045	GSM176046	GSM176047
GSM176048	GSM176049	GSM176050	GSM176051	GSM176052	GSM176053
GSM176054	GSM176056	GSM176058	GSM176059	GSM176060	GSM176061
GSM176064	GSM176065	GSM176066	GSM176067	GSM176068	GSM176069
GSM176070	GSM176071	GSM176073	GSM176077	GSM176116	GSM176120
GSM176124	GSM176125	GSM176147	GSM176148	GSM176149	GSM176150
GSM176151	GSM176152	GSM176153	GSM176154	GSM176155	GSM176156
GSM176157	GSM176158	GSM176159	GSM176160	GSM176161	GSM176162
GSM176163	GSM176164	GSM176165	GSM176166	GSM176167	GSM176168
GSM176169	GSM176170	GSM176171	GSM176172	GSM176173	GSM176174
GSM176175	GSM176176	GSM176177	GSM176178	GSM176179	GSM176180
GSM176181	GSM176182	GSM176183	GSM176184	GSM176185	GSM176206
GSM176207	GSM176210	GSM176211	GSM176212	GSM176213	GSM176214
GSM176215	GSM176216	GSM176217	GSM176222	GSM176223	GSM176224
GSM176225	GSM176226	GSM176233	GSM176293	GSM176295	GSM176296
GSM176297	GSM176344	GSM176345	GSM176346	GSM176347	GSM176352
GSM176353	GSM176354	GSM176355	GSM176357	GSM176363	GSM176364
GSM176365	GSM176366	GSM176367	GSM176369	GSM176370	GSM176371
GSM176372	GSM176373	GSM176374	GSM176379	GSM176380	GSM176381
GSM176382	GSM176383	GSM176384	GSM176393	GSM176394	GSM176395
GSM176397	GSM176398	GSM176401	GSM176402	GSM176403	GSM176404
GSM176411	GSM176412	GSM176413	GSM176436	GSM176445	GSM176446
GSM176447	GSM176448	GSM176451	GSM176452	GSM176453	GSM176454
GSM184354	GSM184355	GSM184356	GSM184357	GSM184358	GSM184359
GSM184360	GSM184361	GSM184362			
