# Supplementary material for: Evaluation of Candidate Genes from Orphan FEB and GEFS+ Loci by Analysis of Human Brain Gene Expression Atlases
Source: PLoS One. 2011 Aug 17;6(8):e23149. doi: 10.1371/journal.pone.0023149 (PMC3157479; doi:10.1371/journal.pone.0023149)
Supplement: Table S2 — Results of the leave-one-out tests for human CNS-related OMIM phenotypes. Legend: represents the size of the artificial loci having a maximum of 2+1 genes. The average numbers of effective candidates with expression profiles and the numbers of evaluated – pairs are shown. The observed and expected numbers of – pairs, for which the true phenotype-causing gene ranks first, among the top ten and within the best 10% of the prioritized list, is reported along with the corresponding -values (one-tailed Fisher exact test). Significant -values are highlighted ( 0.05; 0.01; 0.001). Reference genes (Ref.) were either taken from similar phenotypes (sim.), or from the OMIM disease phenotype itself (dis.). Results taken from [18]; artificial loci with = 400 have not been evaluated in our previous study. (PDF) [file pone.0023149.s002.pdf]

Evaluation of candidate genes from orphan FEB and GEFS+ loci by analysis of human brain gene expression atlases

Rosario M. Piro, Ivan Molineris, Ugo Ala
and Ferdinando Di Cunto

Table S2

Results of the leave-one-out tests for human CNS-related OMIM phenotypes (see the next page).

Expression data	Ref.	N	Candidates (average)	g–p pairs	Ranked first			Ranked 1st–10th			Ranked $\leq$ 10%		
					Obs.	Exp.	P-value	Obs.	Exp.	P-value	Obs.	Exp.	P-value
Mouse Brain Atlas†	sim.	50	73.8	797	16	11	7.95e-02	149	108	2.64e-05***	126	80	1.80e-07***
Mouse Brain Atlas†	sim.	100	137.7	844	13	6	9.84e-03**	105	61	6.26e-08***	132	84	1.93e-07***
Mouse Brain Atlas†	sim.	200	256.6	847	6	3	1.16e-01	61	33	4.84e-06***	139	85	5.08e-09***
Human Brain Atlas	sim.	50	65.9	675	17	10	3.05e-02*	141	102	3.91e-05***	97	68	1.81e-04***
Human Brain Atlas	sim.	100	121.4	745	14	6	4.06e-03**	105	61	5.43e-08***	111	75	1.53e-05***
Human Brain Atlas	sim.	200	224.2	748	8	3	2.04e-02*	54	33	4.44e-04***	117	75	9.32e-07***
Human Brain Atlas	sim.	400	387.4	748	6	2	1.39e-02*	35	19	6.93e-04***	116	75	1.61e-06***
Human Brain Atlas	dis.	50	67.1	188	12	3	2.45e-05***	53	28	1.78e-06***	41	19	1.15e-06***
Human Brain Atlas	dis.	100	124.7	209	10	2	7.82e-06***	46	17	2.46e-10***	49	21	1.09e-08***
Human Brain Atlas	dis.	200	227.4	211	7	1	4.51e-05***	29	9	6.01e-08***	51	21	1.98e-09***
Human Brain Atlas	dis.	400	387.0	211	3	1	1.77e-02*	19	5	2.97e-06***	50	21	6.01e-09***
GEO dataset	sim.	50	89.4	912	29	10	7.65e-07***	190	102	2.08e-17***	163	91	2.26e-13***
GEO dataset	sim.	100	170.5	926	24	5	2.71e-09***	115	54	5.03e-14***	170	93	7.54e-15***
GEO dataset	sim.	200	316.7	929	18	3	2.01e-09***	82	29	2.25e-16***	176	93	1.37e-16***
GEO dataset	sim.	400	549.4	929	11	2	1.59e-06***	51	17	9.25e-12***	170	93	1.78e-14***

N represents the size of the artificial loci having a maximum of $2N+1$ genes. The average numbers of effective candidates with expression profiles and the numbers of evaluated g–p pairs are shown. The observed and expected numbers of g–p pairs, for which the true phenotype-causing gene g ranks first, among the top ten and within the best 10% of the prioritized list, is reported along with the corresponding P-values (one-tailed Fisher exact test). Significant P-values are highlighted ($^{*}P<0.05$; $^{**}P<0.01$; $^{***}P<0.001$). Reference genes (Ref.) were either taken from similar phenotypes (sim.), or from the OMIM disease phenotype itself (dis.). †Results taken from Piro et al., *Bioinformatics* 26:i618–i624, 2010; artificial loci with N=400 have not been evaluated in our previous study.
