# Supplementary material for: Evaluation of Candidate Genes from Orphan FEB and GEFS+ Loci by Analysis of Human Brain Gene Expression Atlases
Source: PLoS One. 2011 Aug 17;6(8):e23149. doi: 10.1371/journal.pone.0023149 (PMC3157479; doi:10.1371/journal.pone.0023149)
Supplement: Table S3 — Overlap of correct leave-one-out predictions (over all CNS-related OMIM phenotypes) for reference genes from similar phenotypes. Legend: represents the size of the artificial loci, having a maximum of 2+1 genes. The number of predictions with the true phenotype-causing gene ranking first, among the top ten and within the best 10% of the prioritized list (see also Table S2, is reported for the Human Brain Atlas (HBA) and the GEO microarray dataset (GEO) along with their overlap. Parentheses indicate the corresponding fraction of the maximum possible overlap. (PDF) [file pone.0023149.s003.pdf]

Evaluation of candidate genes from orphan FEB and GEFS+ loci by analysis of human brain gene expression atlases

Rosario M. Piro, Ivan Molineris, Ugo Ala
and Ferdinando Di Cunto

Table S3

Overlap of correct leave-one-out predictions (over all CNS-related OMIM phenotypes) for reference genes from similar phenotypes.

N	Ranked first			Ranked 1st–10th			Ranked $\leq$ 10%		
	HBA	GEO	overlap	HBA	GEO	overlap	HBA	GEO	overlap
50	17	29	0 (0%)	141	190	37 (27.6%)	97	163	23 (23.7%)
100	14	24	0 (0%)	105	115	14 (16.3%)	111	170	27 (24.3%)
200	8	18	0 (0%)	54	82	4 (7.4%)	117	176	30 (25.9%)
400	6	11	0 (0%)	35	51	2 (5.9%)	116	170	28 (24.8%)

N represents the size of the artificial loci, having a maximum of $2N+1$ genes. The number of predictions with the true phenotype-causing gene g ranking first, among the top ten and within the best 10% of the prioritized list (see also Table S2), is reported for the Human Brain Atlas (HBA) and the GEO microarray dataset (GEO) along with their overlap. Parentheses indicate the corresponding fraction of the maximum possible overlap.
