# Supplementary material for: Evaluation of Candidate Genes from Orphan FEB and GEFS+ Loci by Analysis of Human Brain Gene Expression Atlases
Source: PLoS One. 2011 Aug 17;6(8):e23149. doi: 10.1371/journal.pone.0023149 (PMC3157479; doi:10.1371/journal.pone.0023149)
Supplement: Text S1 — Supplementary Methods. Definition of CNS-related Mendelian disorders; similarity of human disease phenotypes; leave-one-out cross validation. (PDF) [file pone.0023149.s005.pdf]

Evaluation of candidate genes from orphan FEB and GEFS+ loci by analysis of human brain gene expression atlases

Rosario M. Piro, Ivan Molineris, Ugo Ala
and Ferdinando Di Cunto

Supporting Text S1 (Supplementary Methods)

1 CNS-related Mendelian disorders

For the leave-one-out cross validation (LOOCV), we used the same information on human Mendelian disease phenotypes as in our previous study [6] on the Allen Mouse Brain Atlas [1], such that we could compare the results from the two studies. We obtained data from OMIM [2, 3] on 17 June 2009, considering only the 749 phenotype entries of known molecular basis (OMIM symbol: #) containing the term 'central nervous system' in their Clinical Synopsis section. We downloaded the lists of known associated disease genes (mim2gene) from Entrez Gene [3] on 16 June 2009. Between 1 and 25 genes (on average 1.3 genes) were associated to each OMIM phenotype ID; only six phenotypes (<1%) had 10 or more associated genes.

2 Similarity of human disorders

To measure the pairwise similarity of OMIM phenotype entries, we processed the textual descriptions of all OMIM phenotype entries (not limited to CNS-related disorders) using MimMiner, essentially as described by van Driel et al. [4].

MimMiner scores are normalized and range from 0 (unrelated) to 1 (highly related or identical). Since it was established that similar phenotypes can be identified with reasonable accuracy considering a minimum score of 0.4 [4], we used the same threshold for our work.

Using a notion of phenotype similarity allows to select reference genes also for phenotypes with so far unknown molecular basis or increase the number of reference genes for phenotypes with known molecular basis by taking reference genes known to be involved in similar phenotypes.

3 Leave-one-out

We performed large-scale LOOCVs for the spatially mapped expression data from the HBA and for the GEO dataset as previously described [6] and briefly summarized as follows:

For each known gene–phenotype (g–p) pair, we constructed an artificial locus comprising g and the N closest genes on both sides of the chromosome (containing thus $2N+1$ genes centered around g, or less for g close to a chromosome terminal). We chose four representative sizes for artificial loci (N=50, N=100, N=200, and N=400 with a maximum number of 101, 201, 401, and 801 positional candidates, respectively) and determined the lists of positional candidates (in terms of Entrez gene IDs) within these loci from the UCSC Genome Browser [5].

As candidate genes we considered those genes within the artificial locus for which expression data was available (simulating an 'orphan' locus obtained by linkage analysis or comparable techniques), applied the prioritization method as described in [6], and recorded the relative rank/position $\mathcal{R}_g^{rel}$ of the phenotype-causing gene g among the prioritized positional candidates from the artificial locus:

$$\mathcal{R}_g^{rel} = \frac{\mathcal{R}_g}{|C_p|} \qquad \text{with} \quad 1 \leq \mathcal{R}_g \leq |C_p| \Rightarrow 0 < \mathcal{R}_g^{rel} \leq 1 \tag{1}$$

where $\mathcal{R}_g$ is the rank of g within the prioritized genes and $|C_p|{\leq}2N{+}1$ is the number of 'effective' candidates for which expression data was available.

However, we limited the analysis to gene–phenotype pairs having corresponding artificial loci with $|C_p|{\geq}50$. We reasoned that a lower number of effective candidate genes that can be evaluated would introduce an undesired bias by automatically placing the true phenotype-causing gene in higher ranks. Also, we required g itself to have expression data (i.e. $g{\in}C_p$).

As reference genes for the LOOCVs we either took all genes known to be involved in the given OMIM phenotype (excluding g itself)—to simulate phenotypes with already partly known molecular basis—or all genes known to be involved in OMIM phenotypes similar to p (excluding p itself)—to simulate phenotypes of so far unknown molecular basis. For the simulation of phenotypes with known molecular basis at least two known disease genes were required (one taken as candidate and one as reference gene).

References

[1] Lein,E.S. *et al.* (2007) Genome-wide atlas of gene expression in the adult mouse brain. *Nature*, **445**, 168–176.

[2] Amberger,J. *et al.* (2009) McKusick's Online Mendelian Inheritance in Man (OMIM). *Nucleic Acids Res.*, **37**, D793–D796.

[3] Sayers,E.W. *et al.* (2010) Database resources of the National Center for Biotechnology Information. *Nucleic Acids Res.*, **38**, D5–D16.

[4] van Driel,M.A. *et al.* (2006) A text-mining analysis of the human phenome. *Eur. J. Hum. Genet.*, **14**, 535–542.

[5] Kent,W.J. *et al.* (2002) The human genome browser at UCSC. *Genome Res.*, **12**, 996–1006.

[6] Piro,R.M. *et al.* (2010) Candidate gene prioritization based on spatially mapped gene expression: an application to XLMR. *Bioinformatics*, **26**, i618–i624.
